# *Loa loa* in the Vitreous Cavity of the Eye: A Case Report and State of Art

**DOI:** 10.4269/ajtmh.22-0274

**Published:** 2022-08-01

**Authors:** Elisabetta Pallara, Sergio Cotugno, Giacomo Guido, Elda De Vita, Aurelia Ricciardi, Valentina Totaro, Michele Camporeale, Luisa Frallonardo, Roberta Novara, Gianfranco G. Panico, Pasquale Puzo, Giovanni Alessio, Sara Sablone, Michele Mariani, Giuseppina De Iaco, Eugenio Milano, Davide F. Bavaro, Rossana Lattanzio, Giulia Patti, Roberta Papagni, Carmen Pellegrino, Annalisa Saracino, Francesco Di Gennaro

**Affiliations:** ^1^ Clinic of Infectious Diseases, Department of Biomedical Sciences and Human Oncology, University of Bari “Aldo Moro,” Bari, Italy;; ^2^ Section of Ophthalmology, Department of Medical Science, Neuroscience and Senso Organs, Bari Policlinico Hospital University of Bari, Bari, Italy;; ^3^ Section of Legal Medicine, Department of Interdisciplinary Medicine, University of Bari “Aldo Moro,” Bari, Italy

## Abstract

*Loa loa* is a filarial nematode responsible for loiasis, endemic to West–Central Africa south of the Sahara and transmitted by flies. This study reports a case of *L. loa* in the vitreous cavity of the eye of a young patient, along with an in-depth literature review. A 22-year-old woman from Cameroon who migrated from Cameroon to Italy was referred to the Emergency Ophthalmology Department at Policlinico di Bari in July 2021 with the presence of a moving parasite in the subconjunctiva of the left eye. A recent onset of a papular lesion on the dorsal surface of the right wrist and a nodular lesion in the scapular region were detected. *L. loa* filariasis was diagnosed based on anamnestic data, clinical and paraclinical signs, and a parasitological test confirming the presence of microfilariae in two blood samples collected in the morning of two different days. Because of the unavailability of diethylcarbamazine (DEC), albendazole (ALB) 200 mg twice daily was administered for 21 days. A mild exacerbation of pruritus occurred during treatment, but resolved with the use of an antihistamine. A single dose of 12 mg ivermectin was prescribed at the end of the treatment with albendazole. Unlike other endemic parasite infections, *L. loa* is not included in the Global Program to Eliminate Lymphatic Filariasis, because it is not mentioned in the WHO and CDC list of neglected tropical diseases. This can result in an overall risk of lack of attention and studies on loiasis, with lack of data on global burden of the disease.

## INTRODUCTION

*Loa loa*, a filarial nematode called the African eye worm, is responsible for loiasis, endemic to West–Central Africa south of the Sahara, and transmitted by deer flies in the genus *Chrysops* spp. Several local names, such as *yolo li* (*yolo* = worm; *li* = eye) and *guildé guité* (*guildé* = worm; *guité* = eye) are used by the different ethnic groups in eastern Cameroon for referring to the disease.^1^ Patients affected by loiasis belong to two different groups: patients from endemic areas and patients with a history of travel to endemic areas. The geographic distribution of *L. loa* is restricted to central African countries, where the main vectors, *Chrysops silacea* and *Chrysops dimidiata*, occur. As the etymology indicates, the adult filarial localization is in the subconjunctiva of the eye, along with the typical manifestation called Calabar swelling, which may involve different body sites. History of the patient is fundamental in suspected cases in nonendemic regions, and microfilaremia in blood smears is recommended for correct diagnosis and proper treatment. Evidence of *L. loa* in the eyes is transitory, and other symptoms could mimic an allergic reaction, making diagnosis often difficult. We report a case of *L. loa* in the vitreous cavity of the eye in a young patient from Cameroon, and provide an in-depth literature review.

## CASE REPORT

**Figure 1.**
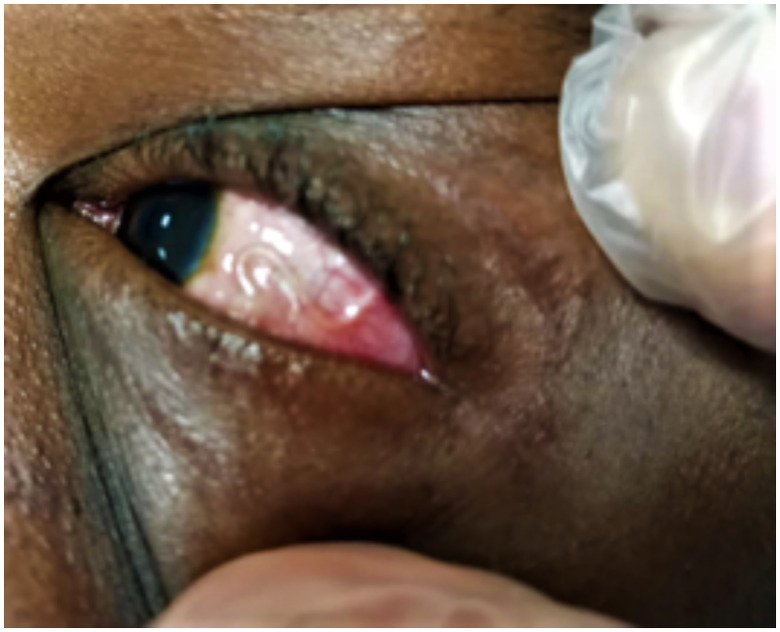
*Loa loa* in the patient’s eye during the first access at the Ophthalmology Unit. This figure appears in color at www.ajtmh.org.

In August 2021, a 22-year-old woman from Cameroon who had been living in Italy for 4 months was referred to the Emergency Ophthalmology Department at Policlinico di Bari with photophobia, tearing, and itching in the left eye. Her medical history included abdominal discomfort for a year, dysmenorrhea with a history of polycystic ovaries, spontaneous abortion in January 2021, and fracture of the orbit floor that required hospitalization in July 2021. She reported a migration route started in 2017 through Nigeria, Niger, Algeria, and Libya where she lived for 18 months. Upon arrival at the Emergency Ophthalmology Department, the presence of a moving parasite in the subconjunctiva of the temporal region was observed during the clinical examination (Figure 1) with concomitant conjunctival hyperemia and eyelid edema, without ulceration. Therefore, no subcutaneous serpiginous cord was observed, but a recent onset of a 0.7-cm papular lesion on the dorsal surface of the right wrist and a nodular lesion of 0.5 × 1.5 cm in the scapular region were detected. The orbital and head computed tomographic scan was negative, whereas the total body computed tomographic scan showed bilateral axillary lymphadenopathy. Because filariasis was at first suspected, she was admitted to the Ophthalmology Unit for surgical removal, but the following day the worm was no longer visible in the eye. Therefore, based on suspected loiasis, the patient was moved to the infectious disease ward where, on physical examination, the patient mentioned cutaneous itching and abdominal pain to the lower abdominal wall. The eye parasite was not visible throughout the patient’s hospitalization despite a foreign-body sensation in the eye and conjunctival hyperemia. The complete blood cell count showed eosinophilia (18%); moreover, a high level of IgE (320 U/mL) was detected, in the absence of other abnormalities. In additions, the patient scored positive for anti-hepatitis B surface and anti-hepatitis B core antibodies. On microscopic examination of blood collected during daylight hours, microfilariae were observed. *L. loa* filariasis was diagnosed based on anamnestic data (i.e., area of origin: Cameroon), clinical (i.e., presence of parasite in the eye, pruritus) and paraclinical signs (i.e., eosinophilia and hyper-IgE), and parasitological test confirming the presence of microfilariae (MF) in two blood samples collected in the morning of two different days (at 8:00 am and 11 am, respectively) showing a different parasitic load of 144 MF/mL and 460 MF/mL, respectively. No indication for biopsy of the previously described cutaneous lesions was given by the dermatologist. Because of the unavailability of diethylcarbamazine (DEC), albendazole (ALB) 200 mg twice daily was administered for 21 days. A mild exacerbation of pruritus occurred during treatment and resolved with the use of an antihistamine. A single dose of 12 mg ivermectin (IVM) was prescribed at the end of the treatment with albendazole. During treatment, an ocular ultrasound was performed and showed negative results. After 1 week of antiparasitic treatment, a microfilaremia reduction was detected (30 MF/mL) along with a progressive decrease of conjunctival hyperemia and eyelid edema. Only a 2-, 4-, and 6-week follow-up after the end of treatment was possible because of the patient’s relocation to another region. Her prognosis was good, and she not referred for any symptoms associated with the illness.

## EPIDEMIOLOGY

**Figure 2.**
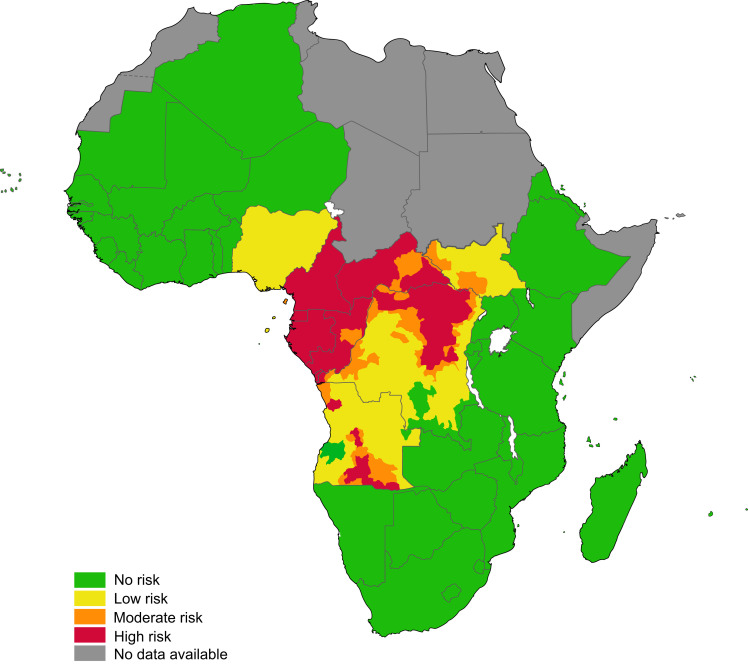
Country map indicating the risk of *Loa loa* infection This figure appears in color at www.ajtmh.org.

The distribution of *L. loa* is restricted to Africa, mainly from southeastern Benin in the west to southern Sudan and Uganda in the east, and from Chad in the North to Zambia in the south. A greater prevalence of human infection has been reported from savannah areas, mainly in rainforest regions characterized by favorable environmental conditions for *Chrysops* vector development.^2^ Areas within the Democratic Republic of Congo and Cameroon account for almost 40% of the population at risk of *L. loa* infection. An estimated 14 million individuals reside in high-risk areas, where the prevalence of eye worm infection is greater than 40%.^2^ Literature data describe cases of ocular filariasis by *L. loa* in patients native to endemic areas and in visitors to endemic areas. In the latter, the clinical manifestations are more likely characterized by Calabar swelling, and more pronounced eosinophilia^3^^,^^4^ may be related to genetic reasons, number of bites received, visitor age and length of infection. Travelers are more likely to be infected when they are bitten by flies for many months, but occasionally people were infected even if the stay in the endemic area was shorter than 30 days.^5^ The burden posed by loiasis is probably underestimated, although 3 to 13 million people are at risk of infection by this filarial nematode.^6^ Figure 2 shows the country map of *L. loa* infection distribution.

## LIFE CYCLE

The parasite is transmitted by vectors belong to the *Chrysops* genus (order, Diptera; family, Tabanidae), with *C. silacea* and *C. dimidiata* being the species most involved. *C. silacea* (Austen) is certainly the most important vector of *L. loa* in humans because of its biting attitude and the capacity of *L. loa* development. These tabanid flies are commonly present in houses, because they are attracted by wood smoke. They ingest microfilariae during the blood meal on infected humans during daylight hours.^7^ After ingestion, the microfilariae lose their sheath, develop into first-stage larvae, and subsequently into third-stage infective larvae in the thoracic muscles of the fly. These latter move to the fly’s proboscis, ready to infect another human host. The development into L3 larvae is temperature dependent and takes 10 to 12 days at 20 to 30°C—temperatures typical of the rainforest zone on the Niger Delta, or about 3 to 4 weeks at the lower temperatures in the mountain valleys and forest fringe in the British Cameroons.^8^^,^^9^ *C. silacea* is also known as “red fly”’ for its orange abdomen with black stripes, can be distinguished from *C. dimidiata* by its brighter colors and tighter stripes. Geographic distribution of these two kinds of fly is almost comparable.^11^

**Figure 3.**
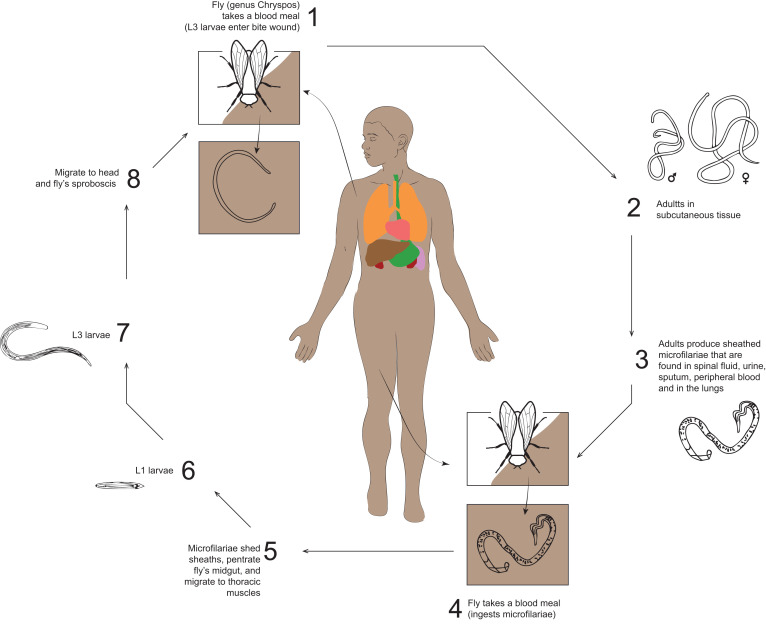
*Loa loa* life cycle. This figure appears in color at www.ajtmh.org.

*Chrysops* spp. tend to bite humans during the daytime, transmitting L3 larvae during blood meals and becoming adults in 150 to 170 days in the subcutaneous tissues. Occasionally, they migrate to the eye.^8^ Mated female worms release microfilariae circulating in the bloodstream during the daytime and, although they are not responsible for direct symptoms of loiasis, they may contribute to complications. Microfilariae can also be recovered from urine and spinal fluid. Each adult worm can produce between 12,000 larvae and 39,000 larvae,^12^ and their life span can reach 20 years (Figure 3).

## CLINICAL MANIFESTATIONS

**Figure 4.**
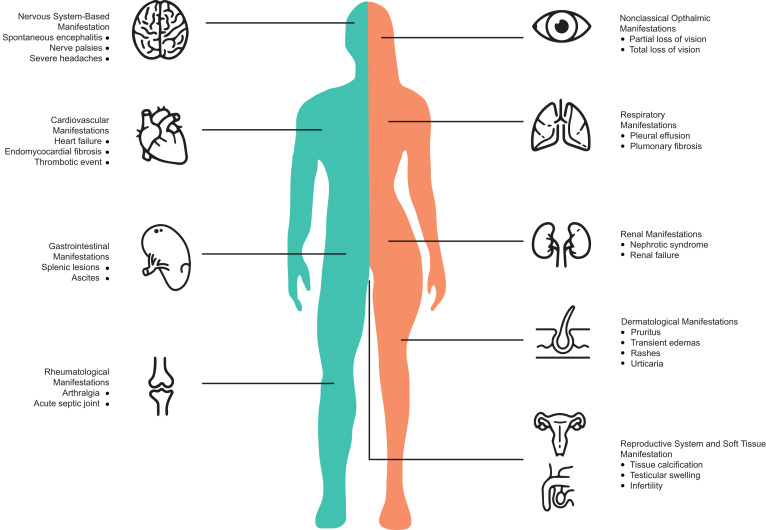
Clinical manifestations of loiasis. This figure appears in color at www.ajtmh.org.

Infected people may remain asymptomatic for days or even years, and symptoms are often benign and transient.^6^ The main clinical manifestations are ophthalmological signs, such as foreign-body sensation, and pain and pruritus in the eye with an occasional decrease in vision resulting from a subconjunctival or conjunctival presence of the worm.^13^ Other systemic manifestations (Figure 4) may include fever, fatigue, itchy legs, and a transient angioedema called Calabar swelling occurring mainly on the ankles, wrists, or face, and especially surrounding the eyes.^14^ The adult worm might disappear through the upper fornix if not removed immediately after eye examination; however, adopting a facedown position may help the worm reappear in the eye.^15^ Allergic manifestations, both cutaneous or visceral, are generally associated with worm rupture in presence of a heavy parasite load, after proper treatment, or spontaneously, although less frequently.^13^ Loiasis may cause glomerulonephritis mediated by immunological injury, as occurs in other more common parasitic diseases such as malaria and schistosomiasis. In this case, a kidney biopsy may show globally sclerotic glomeruli and proteinuria from a urine test. Global renal function might be preserved or might need hemodialysis with correction of DEC posology.^16^ Heart involvement may rarely occur with endomyocardial fibrosis related to long periods of eosinophilia during *L. loa* infection.^17^^,^^18^ Possible complications of ophthalmological involvement resulting from the presence of an adult worm in the anterior chamber could be chronic uveitis, cataract, glaucoma, and corneal edema. An accidental case of *L. loa* infection in bone marrow was described in Canada in a 57-year-old patient from Gabon with T-cell leukemia during workup; microfilaria with nuclei extending to the tip of the tail, consistent with an *L. loa *diagnosis, were found in a bone marrow biopsy, then confirmed with Giemsa stain on peripheral blood and on marrow biopsy.^19^ Clinical complication and disease severity may depend on patient age and time of diagnosis.^20^^,^^21^

Evidence from the literature report typical neurological manifestations such as encephalitis, along with consciousness, asthenia, headache, chills, blurred vision, and difficulty speaking. Predisposing factors for damage to the blood–brain barrier are high blood concentrations of microfilariae and coexisting infections with *Trypanosoma *spp., *Treponema pallidum*, and *Plasmodium* spp.^22^^,^^23^

Nevertheless the microfilariae burden as a predisposing risk factor for encephalitis is still debatable. Indeed, most cases of loiasis-associated encephalitis are reported with a load > 30,000 MF/mL in the blood, although neurological manifestations were also observed in patients with lower microfilariae blood concentrations.^22^ Neurological and psychiatric sequelae have been described among the survivors.

## DIAGNOSIS

The diagnosis of loiasis is a challenge for clinicians for several reasons. One of the main typical symptoms is the foreign-body sensation and eye swelling. Thus, ophthalmologists are often the first specialists who face this neglected disease. Nevertheless, slit lamp examination or naked-eye observation is not always diagnostic because of the fast migration of the worm to the retrobulbar space, which often occurs before the clinical examination. In a single case report, the use of anterior segment optical coherence tomography as an alternative imaging tool revealed an adult worm in a 9-year-old child from Equatorial Guinea.^24^ Similarly, ultrasonography of the right calf (Calabar swelling) revealed a pipeline‐shaped lesion suggestive of nematode presence; naked-eye examination was unable to detect it.^25^

Furthermore, the blood detection of microfilariae represents one of the main diagnostic tools for the management of loiasis for clinicians to define a proper therapeutic strategy. An appropriate blood sample that depends on the life cycle of parasites and insect behavior, which varies among the different agents of filariasis, needs to be considered. *L. loa* microfilariae are detected primarily in the blood in the daytime (i.e., between 10:00 am and 2:00 pm), when the insect feeds on the host.^26^

In addition to timing, the CDC provides guidelines for the correct morphological identification of microfilariae detected by blood smear.^5^ In particular, *L. loa* microfilariae are 231 to 250 μm in length, with a relatively short headspace and a tail with nuclei irregularly arranged to the tapered tip; their typical sheathed morphological feature is colorless via Giemsa stain.^26^ Recently, a description of three types of cuticular worms was observed first by scanning electron microscopy for differentiating *L. loa* from other filarial nematodes.^27^

Microfilaremia may be intermittent, therefore laboratory parameters, such as eosinophilia, are useful when suspecting infection (Table 1).

**Table 1:** Diagnostics of loiasis: features of test

Diagnostic test	Function	Strength	Weakness
Naked eye examination/slit lamp eye examination	Eye involvement	Surgical excision possible Direct observation with microscopy for certain diagnosis	Not constant observation because of irregular and unpredictable migration of the worm
Imaging with high-resolution computed tomography and ultrasound	Nematode detection if no direct observation is possible	Useful if migration of the worm happens before medical observation	Low sensitivity Few case reports in literature and lack of standards
Microscopic evaluation of blood sample	Treatment decision according to microfilarial load cutoff	Certain diagnosis through morphological evaluation if electronic microscopy is used Cell phone test developed as point-of-care test for mass screening or difficult-to-reach medical center	If no microfilariae are found, diagnosis is not excluded Appropriate timing of sample collection required to increase sensitivity
Serological test	Previous or recent parasite infection Endemicity assessment	Mass screening to prevent to prevent severe adverse effects in mass drug administration campaign against onchocerciasis Positive predictive value in case of no findings during ophthalmological examination and amicrofilaremic cases Rapid antigen test available with same efficacy of ELISA for mass screening or difficult-to-reach medical center	Cross-reactivity with other lymphatic filariases
Rapid assessment procedure for loiasis interview	Endemicity assessment	Mass screening as efficient as serological test Correlation with microfilarial load Low cost	Function limited to screening
Antigen on urine sample	Nematode detection in amicrofilaremic cases	Positive predictive value in case of no findings during ophthalmological examination and amicrofilaremic cases	Still in study
Polymerase chain reaction test on blood sample or on Calabar edema	Nematode detection in amicrofilaremic cases	Positive predictive value in case of no findings during ophthalmological examination and amicrofilaremic cases Correlation with quantification of microfilarial load if loop-mediated isothermal amplification and restriction fragment length polymorphism–polymerase chain reaction are searched.	High cost
Increased eosinophil count	Indirect sign of parasitic infection	Reinforce the suspicion of parasitic infections Marker for treatment follow-up	If absent, diagnosis not excluded
Increased IgE values on serum	Indirect sign of parasitic infection	Reinforce the suspicion of parasitic infections	If absent, diagnosis not excluded

The use of biomarkers for loiasis diagnosis is still debated. Several approaches have been considered using antibodies, antigens, or nucleic acid detection for the development of a diagnostic tool based on a biomarker. For example, serological tests detecting both heterologous and homologous species of antibodies with electro-syneresis and ELISA could not distinguish between active and past infections. Furthermore, cross-reaction among filarial antigens decreases the specificity of the tests. Nevertheless, the review flags *L. loa*-specific Ig4 elevation during loiasis, also among 70% of amicrofilaremic cases, and the seroconversion refers to about 50% of cases. All in all, considering the acceptable sensitivity (54%), a serological test can play a more helpful diagnostic role in nonendemic areas and where there is not a significant prevalence of other parasite infections (e.g., *Onchocerca*, soil-transmitted helminths), although for other filariases the correlation between circulating antigen test and microfilaremia showed good results but poor sensitivity and low reliability for *L. loa*.^28^ In a recent review, Gobbi et al.^29^ compared the performance of a *Loa* antibody rapid diagnostic test and a commercial ELISA pan-filarial test on 170 patients with various parasitic infection (*L. loa*, *Mansonella perstans*, *Brugia* spp., soil-transmitted helminths) reporting high sensitivity of both the rapid diagnostic and pan-filarial serology tests for the diagnosis of loiasis. Meanwhile, the detection of antigen from urine samples through the luciferase immunoprecipitation system is an object of ongoing studies that might lead to new diagnostic tools, although is not still validated.

The use of nucleic acid as a biomarker in loiasis infection has been also investigated. The most promising molecular tests are loop-mediated isothermal amplification and restriction fragment length polymorphism–polymerase chain reaction (PCR), which correlate the parasite DNA to microfilaremic levels and are also useful in amicrofilaremic individuals. Conventional PCR is used for the diagnosis of loiasis, whereas quantitative PCR is useful for the quantification of microfilariae.^28^ Molecular tests play a pivotal role when clinical suspicion is still high, even when first line tests are negative, such as blood sample, microscopic analysis, slit lamp observation of the eye and serological test. Indeed, in a traveler in an endemic country (e.g., Equatorial Guinea) with compatible symptoms of ocular filariasis, in the absence of microfilaremia and adult worms in the eye, a diagnosis has been reached by nested PCR from a biopsy of Calabar swelling.^25^

**Figure 5.**
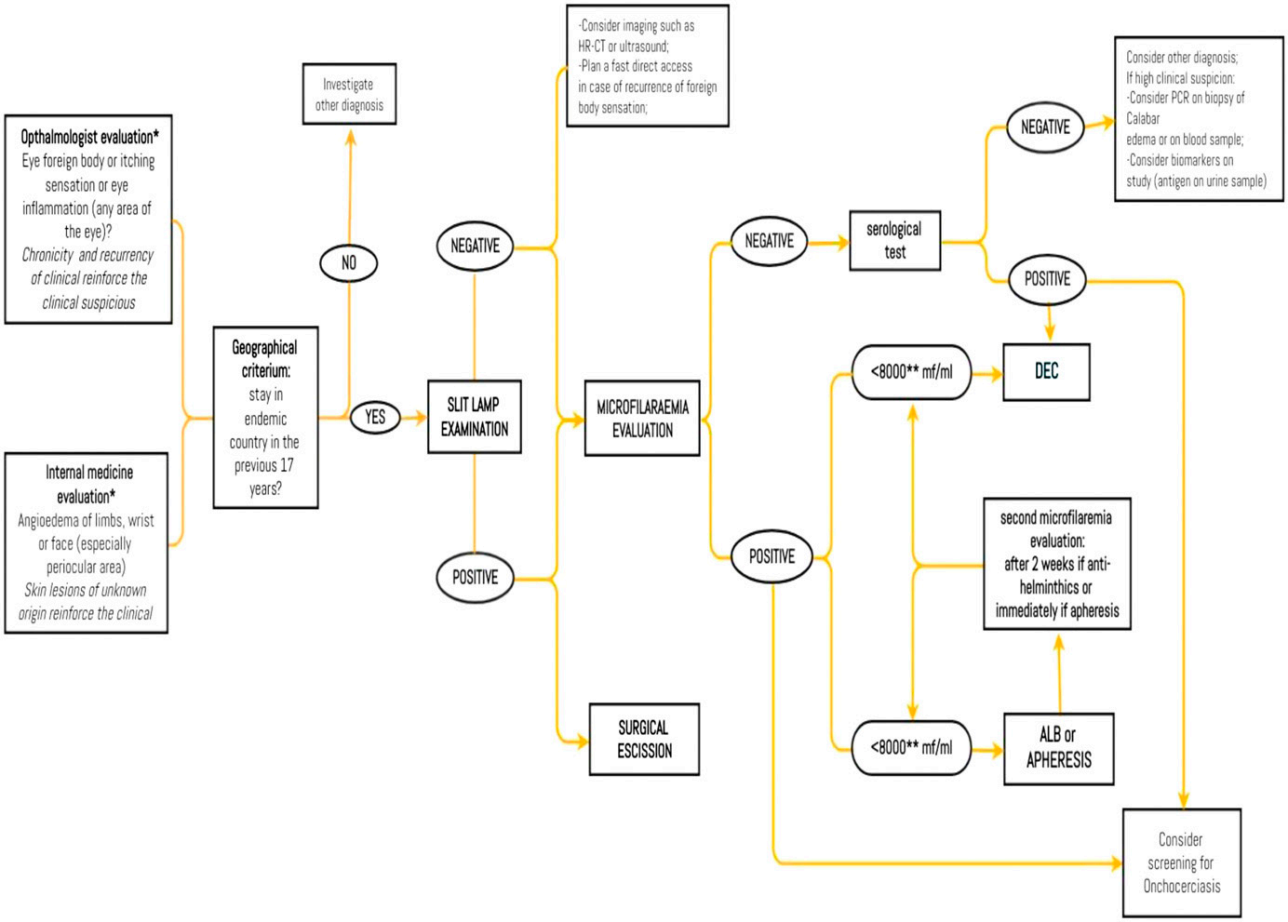
Proposal of flowchart for the diagnosis and treatment of loiasis. *Increased eosinophil count and IgE on serum reinforce the clinical suspicion; but, if absent, diagnosis cannot be excluded. **Some authors suggest a more conservative treatment using 2,500 MF/mL as a cutoff for albendazole. This figure appears in color at www.ajtmh.org.

Therefore, loiasis, like most of the neglected tropical diseases (NTDs), represents an important public health issue—mainly in low-income countries. Thus, clinicians face the challenge of reaching a diagnosis where advanced and standard technologies are not available. Recently, a new system called CellScope combines mobile phone technology with handheld microscope proving an excellent diagnostic efficiency for *L. loa* and *Schistosoma* spp. infection compared with standard diagnostic methods.^30^^,^^31^ Thus, a type of CellScope (LoaScope) has been proposed by World Health Organization (WHO) as a key point-of-care testing tool for NTD elimination in areas with co-endemicity.^10^ In Figure 5, we illustrate our proposal of flowchart of diagnoasis and treatment of loiasis. The diagnosis of loiasis always requires a multidisciplinary approach. Regardless the onset symptoms, both ophthalmologists and infectious disease specialists take part to an accurate wor-up. If the clinic is very suggestive, the serological test is conclusive to make diagnosis, but microfilaeremia count is compelled to choose the best therapeutic option and to reduce the occurrence of DEC-related adverse events. The geographical criterion can be searched in a long temporal arc and once the diagnosis is reached, it is a good practice to screen the patient for the other geographically related NTDs.

## TREATMENT

Treatment of loiasis can be difficult and often requires a strong team collaboration between an expert in infectious diseases or tropical medicine, an ophthalmologist, and a parasitologist. Infection by *L. loa* can remain asymptomatic for months or years, but treatment could be necessary when adult worms cause migratory (Calabar) swelling through the skin or when worms are visible in the eye.^32^

Surgical removement of the live, migratory worm is often needed because one of the most frequent clinical presentations of loiasis is the presence of live worms in the ocular–periocular region.^30^

When still visible under the bulbar conjunctiva, the technique used to remove the worm from the eye often does not resolve the situation because capture of the nematode requires immobilization of the worm.^30^ Removal of the nematode relieves pain and allows confirmation of diagnosis, although histological exam is not essential. Even in cases when the nematode is removed successfully, systemic antimicrobial treatment is a necessity for a cure.^33^

According to the CDC, recommended first-line treatment of systemic loiasis is DEC 8 to 10 mg/kg/d by mouth, divided doses, for 21 days—a highly effective chemotherapeutic that is both macrofilaricidal and microfilaricidal.^32^ Importantly, the administration of this drug in patients with a blood microfilaremia level > 8,000 microfilariae/mL may represent a risk for fatal encephalopathy as a result of lysis of the microfilariae, resulting in the activation of an inflammatory response, so that apheresis procedures are needed to decrease the circulating microfilaremia.^34^ Despite of the use of gradually increasing doses, the use of DEC is characterized by many adverse events (including itching, rash, edema, headache, and fever). But, as state earlier, the most severe reaction is encephalopathy often accompanied by retinal hemorrhage in patients with very high loads, and it is often fatal, with most survivors developing serious sequelae.^10^ These findings often were caused by the massive release of parasite antigens after the rapid death of *L. loa* microfilariae.^30^ Current views suggest that either pharmacological (albendazole) or mechanical (cytapheretic) means should be used to decrease microfilariae counts to < 8,000 microfilariae/mL before the initiation of DEC,^35^ as showed in Table 2. Although only case reports and case series are available, filaria apheresis appears to be a safe and efficacious technique to reduce parasite counts in patients with loiasis. Moreover, albendazole and apheresis appear to reduce DEC-associated neurological complications effectively in a small number of published cases.^32^ The effects of apheresis have been studied since 1983, when Muylle et al.^38^ reported three apheretic sessions with a successful reduction of parasitemia before DEC treatment. ALB is a benzimidazole derivative with a great absorption after oral administration and a wide spectrum of activity.^37^ There are currently no studies that suggest the use of albendazole as an alternative regimen for loiasis, and a short regimen with ALB has been demonstrated to have little effect on microfilaraemia.^38^ Its role is accepted by the CDC if a blood tests shows > 8,000 microfilaria/mL, at a dosage of 200 mg twice daily for 21 days to reduce the *Loa loa* microfilariemia (LLM) and to avoid the neurological adverse effects of DEC. Moreover, given at this dose it seems to have an embryotoxic effect and maybe also a macrofilaricidal effect.^39^

**Table 2:** Pharmaceuticals used in loiasis treatment

Drug	Dosage	Indications	Micro-/ macrofilaricide	Common adverse effects	Contraindications	Guidelines
Diethylcarbamazine	8–10 mg/kg/d by mouth, divided doses per 21 days	Symptomatic loiasis with MF/mL < 8,000	Both	Headache, dizziness, nausea, fever or inflammatory reaction to death of adult worms; encephalopathy	Onchocerciasis, or in patients in whom onchocerciasis cannot be ruled out	Yes
Albendazole	200 mg twice daily per 21 days	Symptomatic loiasis, with MF/mL < 8,000 and two failed rounds diethylcarbamazine; or symptomatic loiasis, with MF/mL ≥ 8,000 to reduce level to < 8,000 prior to treatment with diethylcarbamazine*	Macro	Headache, increased liver-associated enzymes	–	Yes
Apheresis followed by diethylcarbamazine	–	Symptomatic loiasis, with MF/mL ≥ 8,000	–	–	–	Yes
Ivermectin	150 µg/kg, single dose	Co-infection with onchocerciasis	Micro	Fever, pruritic rash; inflammatory reactions to death of adult worms	Loiasis with MF/mL > 5,000	No
Imatinib (tyrosine kinase inhibitor)	–	Alternative to standard care	Macro	Upset stomach, nausea, vomiting, diarrhea, headache, muscle and joint pain, muscle cramps, dizziness, blurred vision, drowsiness	–	No
Prednisone	Start with 60 mg/d	Reduce the intensity of diethylcarbamazine’s adverse events	–	Headache, dizziness, difficulty falling asleep, inappropriate happiness, personality changes, acne	–	No
Reslizumab (anti- interleukin 5)	–	Reduce the intensity of diethylcarbamazine’s adverse events	–	Creatine phosphokinase elevation	–	No

MF = microfilariae of *Loa loa*.

* Some authors suggest a more conservative treatment using 2,500 MF/mL as a cutoff for albendazole.

Recently, a double-blind placebo-controlled trial^40^ with three parallel treatment arms concluded that a six-dose, 800-mg regimen with ALB every 2 months reduced *L. loa* microfilaremia, as ≈50% of the participants experienced a sustained LLM decrease by > 50%.

However, it has been observed that in some cases, patients treated with DEC continued to experience symptoms, as it is curative in about 60% of cases. In patients with persistence of clinical evidence, ALB can be used with success.^32^ IVM is not indicated in the treatment of *L. loa*; some patients who have been exposed to its administration developed serious neurological adverse events after treatment—in particular, in those countries where it was used during elimination strategies of onchocerciasis.^41^ In particular, Wanji et al.^42^ reported that the microscopic and macroscopic changes in treated animals were similar to those noticed in humans who were administered IVM, consisting of microscopic and macroscopic changes resulting from an inflammatory process involving fibrin deposition on the walls of blood vessels.^42^ IVM is a broad-spectrum anthelmintic drug that, similar to DEC, has rapid microfilaricidal effects that can result in severe adverse events (SAEs),^44^ especially in patients with a high level of microfilaremia, but it is not effective with adult worms. Nevertheless, other authors emphasize how a single dose of IVM (150–200 μg/kg) is very useful at reducing *L. loa* microfilarial densities substantially for at least a year, regardless of the initial level of parasitemia.^45^

New strategies were explored in 2018 by Gobbi et al.,^43^ who compared different drug regimens in a retrospective study and concluded that the combination ALB plus IVM provided a high proportion of parasitological cure and, although these results should be taken with caution, it deserves further research considering the synergy of the mode of action of the two drugs.

New prospects of care are required to avoid the already discussed complications that occur as a result of the standard treatment of *L. loa*. O’Connell et al.^47^ discussed the possibility that tyrosine kinase inhibitors can actually play a role.

They have shown that there are genetic similarities between *Brugia malayi* and *L. loa* because both express Abl-like human protein. This protein can be susceptible to treatment with imatinib, a tyrosine kinase inhibitor that prevents the phosphorylation of Tyrosine-protein kinse ABL (c-Abl), which appears to be detrimental to embryogenesis in the adult female of *B. malayi*, so it may be used to lower the levels of microfilarial densities.^48^

Novel approaches are necessary to investigate the prevention of post-treatment reactions in loiasis in contrast to the use of corticosteroids and antihistamines historically proven to reduce the intensity of these adverse events. For example, some authors explored the role of interleukin-5 eosinophilia in post-DEC reactions by the administration of the humanized anti-interleukin-5 antibody reslizumab.^49^

The management of loiasis differs substantially across specialized travel clinics in Europe. These discrepancies could be a result of different local protocols as well as to (un)availability of the drugs. A harmonization of clinical protocols for the treatment of loiasis is suggested across reference centers for tropical medicine in Europe.^46^

## ELIMINATION STRATEGIES AND INTERVENTIONS: VECTOR CONTROL AND POLICY CHANGES

Unlike other endemic parasite infections, *L. loa* is not included in the Global Program to Eliminate Lymphatic Filariasis, because it is not mentioned in the WHO and CDC list of NTDs. This can result in an overall risk of lack of attention and studies on loiasis, with an accompanying lack of data on the global burden of the disease. In a review, Agbolade et al.^50^ stated that loiasis, together with *M. perstans* infection, represents a relevant cause for medical consultation in endemic areas that frequently results in students who are unable to study and adults who are unable to work. Concerning the burden assessment of the specific disease, the minor gravity of symptoms in respect to other NTD, has not been compensated by the evaluation of the consequence on quality of life, which led to underestimating the socioeconomic burden of loiasis. In this regard, some^51^ denounce that a disability-adjusted life-year evaluation has been officially determined for many chronic conditions (infectious or not), but no efforts have been made to assess loiasis. In addition, others^50^ report a low level of knowledge by health workers and the general population about awareness and transmission modes of infection.

The elimination strategy traditionally consists of two parts: parasite infection eradication through Mass Drug Administration (MDA) and vector control.

### MDA: lights and shadows of active intervention.

MDA cannot be separate from the preparatory work of mapping to define the high endemic region where the risk–benefit ratio is favorable for preemptive and general presumptive treatment. The main risk lies in SAEs, depending on a high microfilarial load or co-infection with *Onchocerca* spp. or lymphatic filariasis microorganisms. For this reason, an accurate co-endemicity mapping results in the best outcome of any MDA campaign.

The major tools to define loiasis endemicity are serology detection and the Rapid Assessment Procedure for Loiasis (RAPLOA). Concerning serology detection, cross-reactivity among different parasites represents the main challenge for health policymakers. For instance, the risk of overestimation of lymphatic filariasis (LF) prevalence (particularly for *W. bancrofti*) could occur as a result of the cross-reactivity of rapid diagnostic immunochromatographic card tests (widely used for LF mapping) with *L. loa* in co-endemic areas.^52^

RAPLOA can be considered a very useful tool for defining the prevalence of loiasis, almost as useful as the serological test. The method consists of an interview with focused questions that investigate anamnesis and symptoms of loiasis, which turns out to be very useful in low-resource settings.^53^^,^^54^ In 2012, Wanji et al.^55^ reported the clear relationship between the prevalence of eye worm history and the prevalence and intensity of *L. loa *microfilaremia, and fixed the threshold of 40% of eye worm history as a sensitive and specific indicator of high-risk communities. These findings contribute to validating the RAPLOA tool. Its validity has been experienced also in coordinated use with a rapid epidemiological assessment (REA) for onchocerciasis.^56^ A study by Wanji et al.^56^ carried out in 10 communities in a forested area of Cameroon reached the conclusion that, in areas of co-endemicity of loiasis/onchocerciasis, the combined use of RAPLOA and REA would be advantageous for national onchocerciasis control programs in terms of time and cost savings. In particular, a survey team administered the questionnaire for RAPLOA and, at the same time, performed an REA, confirming the relationships between RAPLOA and *L. loa* microfilaremia prevalence, and between an REA and *Onchocerca volvulus* microfilaremia prevalence, respectively. For this reason, this procedure has proved to be useful for the control of community-directed treatment (CDT) with IVM.

However, until today, *L. loa* has never been targeted by any specific MDA program, as MDA programs concern other NTDs, such as CDT with IVM for onchocerciasis and LF eradication. For this reason, we can observe just indirect effects over loiasis prevalence. Some authors^57^ tried to measure the relationship between the prevalence of *L. loa* and the number of IVM doses distributed among the population during the CDT with IVM in the CDT IVM population in loiasis co-endemic areas where elimination programs against onchocerciasis and LF were ongoing. They observed a negative correlation between microfilaremia load and the number of IVM intake doses; but still, we must report that their They observed a negative correlation between microfilariemia and the intake dose of IVM with considering microfilariemia as parameter of the burden’s disease reductions and not the total number of cases of loiasis. By the way, the result is enough to affirm that annually repeated CDT IVM programs reduce the risk of neurological and non-neurological post-IVM adverse events.^57^

### Vector control: aspects to consider.

With regard to vector control, three methods have to be considered. First, environmental modification, such as draining, filling, or removing vegetation around breeding sites, has been adopted historically, but still the occurring problem of this choice is that the vector can be spread all over vast wooded and hard-to-reach areas. Second, health operator worked on the direct killing of the vector. In 1963, Williams and Crewe^62^ highlighted the success of a 14-mi^2^ application of dichlorodiphenyltrichloroethane dieldrin, aldrin, and γ-hexachlorocyclohexane, which reduced the presence of infective larvae of *L. loa* in *Chrysops* by 62% by penetrating breeding site mud to a depth of 5 to 15 cm. However, they also noted the difficulties in treating large areas of mud and raised significant concerns about the possible seepage of insecticides into streams, which could create public health problems by adversely affecting other non-target animals and humans. Recently, different insecticide-based larvicides such as temephos (Abate) or biological control agents such as *Bacillus thuringiensis’s* toxin (Bti) that specifically kill dipteran larvae are used, which are sprayed periodically by training local operators. The problem of applying larvicides and adulticides in the hard-to-reach forested site could be faced with new technologies, such as drones using unmanned aerial vehicles. All considered, it is important for health policymakers to consider that environmental actions are effective in the long term because of the slow life cycle of the pathogen, since they cannot ensure a short-term change in the co-endemicity prevalence over filariasis and onchocerciasis eradication programs.^5^ For this reason, other direct vector elimination strategies have been considered. Among these, the development of new trapping larvicides reveals to be a multi-functional system. For example, home-made *Nzi* traps (*Nzi* stands for fly in Swahili), carbon dioxide and octanol traps, or wood fires, and the color of traps work are used. It is remarkable that vector trapping acts on two aspects of vector control. On one hand, it decreases the infection spreading by capturing vectors; on the other hand, it allows health operators to monitor species abundance directly for *Chrysops *spp. This last aspect promises to be more efficient than serology detection or RAPLOA.^52^^–^^61^

In addition, remote-sensing satellite imagery and modeled environmental data are now considered to assess the ecological and climatic aspects of habitats and vector behavior, including the extent of deforestation, to define new predictive factors of the risk in a community.

### Where we are now: new perspectives to overcome adverse effects.

IVM-based MDA has been revised for some years because of the inaccurate process of co-endemicity mapping. Neither RAPLOA/serological testing nor direct vector control have managed to reduce the high prevalence of SAEs in co-endemic areas. Still, many gaps affect the assessment of co-endemic areas. In hard-to-reach sites, loiasis prevalence is mostly unknown, and recorded data about previous CDT IVM campaigns and related effects regarding *L. loa* prevalence are lacking.^58^

For this reason, alternative drug regimens have been studied in a co-endemicity-tailored approach, such as twice-yearly ALB administration plus long-lasting insecticidal nets. This protocol proved to decrease SAE prevalence in co-endemic LF and *L. loa* areas with intermediate loiasis risk (RAPLOA between 20% and 40%).^31^^,^^58^ Currently, the WHO recommends this treatment option approach for MDA as the first choice, no matter which RAPLOA percentage is reported or imagined. Moreover, WHO recommendations are mostly against CDT IVM in onchocerciasis–loiasis–LF co-endemicity.^59^^,^^60^

The point-of-care test based on the new rapid LoaScope opens to a new MDA approach in *L. loa* and onchocerciasis co-endemicity, especially in onchocerciasis hypoendemic areas, where the risk of post-IVM SAEs overcomes the benefit of onchocerciasis preventive chemoprophylaxis. In this context, two test-and-not-to-treat strategies can be adopted: the so-called *Loa*-first strategy or its alternative—the so-called *Onchocerca*-first strategy. The *Loa*-first strategy consists of screening for loiasis first and excluding *Loa*-positive individuals from IVM treatment; then, search for *Onchocerca*-positive results among them and use an alternative regimen with doxycycline. With the *Onchocerca*-first strategy, the population undergoes screening for onchocerciasis and then the clinician decides whether to treat them according to a consequential screening for *L. loa*.^61^^–^^63^ Unfortunately, we do not yet provide a point-of-care test for *Onchocerca* spp. as a sensitive remedy, by considering its rate of false-negative results, since people screened would be excluded from *Onchocerca* preventive chemoprophylaxis.^51^

Last, we remark that as long as MDA programs are one of the major weapons in fighting NTD elimination, the risk of incoming pharmaco-resistance will persist and we are bound to keep developing anti-parasite pharmacology.^46^ Thus, considering the multidimensional approach to the loiasis control program, we firmly state there is no other way than adopting integrated vector management to target simultaneously multiple diseases with shared resource use.^10^

## CONCLUSION

Because loiasis is still overlooked and often misdiagnosed, we could somehow consider it such an NTD, although its spread burdens in a consistent part of population. Furthermore, the absence of loiasis among the WHO official list of NTDs has meant that just partial efforts have been made to face this disease.

Nevertheless, two considerations must be made to reduce the diagnostic delay of loiasis in non-endemic areas. First, travel anamnesis must be referred not to a few months, but to some years earlier, because the course of the infection is long and the clinical appearance can be recurrent and subclinical. For this reason, clinicians from high-income, non-endemic countries have to develop an awareness of this disease in their daily practice with migrant and traveler populations. Second, loiasis must not be considered an exclusive concern of infectious disease specialists. Ophthalmologists must be trained to investigate the disease because they are usually the first specialist to encounter these patients. Fast-track procedures for worm removal must be adopted in ophthalmology units because finding a worm in the eye can be temporary. We firmly stress that loiasis has to be faced by a multidisciplinary team that includes infectious disease specialists (possibly a parasite infection expert), microbiologists, and ophthalmologists. Otherwise, the parasite will be faced in a partial and often ineffective way.

Concerning the workup of loiasis, we suggest a focus on microfilaremia to reduce the risk of treatment-related adverse events. Other laboratory tests must be used, because microfilaremia is not a constant finding, and the current biomarkers and serological tests still lack specificity and sensitivity to differentiate ongoing infection from previous contact. For this reason, a correct general evaluation with epidemiological and anamnestic data often plays the most important role.

Concerning treatment, medications proved to be effective in most cases. However, SAEs such as encephalopathy forced clinicians to investigate new solutions. Blood apheresis has been evaluated as successful in some case reports. Moreover, unfortunately although there are a significant number of immigrants and some the neglected tropical diseases may also affect travelers abroad, the majority of the medicines that are recognized as essential by WHO for the treatment of these diseases are not present in most of referral hospitals.^64^

Last, we stress that an efficient assessment through epidemiological studies and through a disability-adjusted life-year definition should be considered to start a systematic action against loiasis. A specific plan against loiasis (instead of a side section of the Global Program to Eliminate Lymphatic Filariasis) would help to fight against 1) the reduction of onchocerciasis and LF defeat programs; 2) the important subacute health burden over the health system (access to visit), and 3) the consistent relation between loiasis and inability to work or attend school in endemic areas. Elimination programs should include both environmental actions against vectors and drug administration, because the first alone cannot show efficient results in the short term.

To organize economic resources for the program, it is important to consider RAPLOA as effective as a serology test, which reduces costs poor-resource areas. In addition, new technologies such as mobile-based technology for microscopic diagnosis is a promising tool from a point-of-care diagnosis perspective.
